# Associations between cytotoxic T-lymphocyte-associated antigen 4 gene polymorphisms and diabetes mellitus: a meta-analysis of 76 case–control studies

**DOI:** 10.1042/BSR20190309

**Published:** 2019-05-15

**Authors:** Min Chen, ShuMin Li

**Affiliations:** Department of Internal Medicine, Ningbo No. 6 Hospital, Ningbo 315040, Zhejiang, China

**Keywords:** Cytotoxic T-lymphocyte-associated antigen 4 (CTLA-4), Diabetes mellitus (DM), Gene polymorphisms, Meta-analysis

## Abstract

**Background:** Several genetic association studies already investigated potential roles of cytotoxic T-lymphocyte-associated antigen 4 (*CTLA-4*) gene polymorphisms in diabetes mellitus (DM), with inconsistent results. Therefore, we performed this meta-analysis to better assess the relationship between *CTLA-4* gene polymorphisms and DM in a larger pooled population.

**Methods:** PubMed, Embase, Web of Science, and CNKI were systematically searched for eligible studies. Pooled odds ratios (ORs) with 95% confidence intervals (CIs) were calculated to estimate the strength of associations between *CTLA-4* gene polymorphisms and DM in all possible genetic models.

**Results:** A total of 76 studies were finally included in our analyses. Significant associations with susceptibility to type 1 diabetes mellitus (T1DM) were detected for rs231775 (dominant model: *P*=0.008, OR = 0.83, 95%CI 0.73–0.95; recessive model: *P*=0.003, OR = 1.27, 95%CI 1.09–1.50; allele model: *P*=0.004, OR = 0.85, 95%CI 0.77–0.95) and rs5742909 (recessive model: *P*=0.02, OR = 1.50, 95%CI 1.05–2.13) polymorphisms in overall population. Further subgroup analyses revealed that rs231775 polymorphism was significantly associated with susceptibility to T1DM in Caucasians and South Asians, and rs5742909 polymorphism was significantly associated with susceptibility to T1DM in South Asians. Moreover, rs231775 polymorphism was also found to be significantly associated with susceptibility to type 2 diabetes mellitus (T2DM) in East Asians and South Asians.

**Conclusions:** Our findings indicated that rs231775 and rs5742909 polymorphisms may serve as genetic biomarkers of T1DM, and rs231775 polymorphism may also serve as a genetic biomarker of T2DM.

## Introduction

Diabetes mellitus (DM), characterized by chronic hyperglycemia caused by deficiency in insulin secretion or resistance against insulin, is the most prevalent metabolic disorder worldwide, and it currently affects over 350 million people globally [1,2]. So far, the exact underlying pathogenic mechanism of DM is still not fully understood. Nevertheless, the fact that over 100 genetic loci were already found to be correlated with an increased susceptibility to DM by past genome-wide association studies suggested that genetic factors were crucial for the occurrence and development of DM [3,4].

Cytotoxic T-lymphocyte-associated antigen 4 (CTLA-4) is mainly expressed on activated T cells, and it serves a negative regulator of T cell activation and proliferation [5]. Previous studies showed that CTLA-4 could induce T cell tolerance and attenuate T cell mediated immune responses by binding with co-stimulating molecules, B7-1 (CD80) and B7-2 (CD86) [6], and dysfunction of CTLA-4 was demonstrated to be implicated in various autoimmune diseases including type 1 diabetes mellitus (T1DM) [7,8]. Consequently, *CTLA-4* gene polymorphisms were intensively studied with regard to their associations with T1DM [9–12]. Recently, some pilot studies also analyzed potential associations between *CTLA-4* gene polymorphisms and the much more prevalent type 2 diabetes mellitus (T2DM) [13,14]. Nevertheless, whether *CTLA-4* gene polymorphisms were associated with T1DM and T2DM or not remain controversial, especially when they were conducted in different populations. Therefore, we performed the present meta-analysis to pool the data of all relevant studies, and obtain more conclusive results on associations of *CTLA-4* gene polymorphisms with T1DM and T2DM.

## Materials and methods

### Literature search and inclusion criteria

The current meta-analysis was complied with the Preferred Reporting Items for Systematic Reviews and Meta-analyses (PRISMA) statement [15]. Potentially relevant articles were searched in PubMed, Medline, Web of Science, and CNKI using the following key words: ‘Cytotoxic T-lymphocyte antigen 4’, ‘CTLA-4’, ‘polymorphism’, ‘variant’, ‘mutation’, ‘genotype’, ‘allele’, ‘diabetes mellitus’, ‘diabetes’, and ‘DM’. The initial literature search was conducted in October 2018 and the latest update was performed in January 2019. We also screened the reference lists of all retrieved articles to identify other potentially relevant studies.

Included studies should met all the following criteria: (1) case–control study on associations between *CTLA-4* gene polymorphisms and individual susceptibility to DM; (2) provide adequate data to calculate odds ratios (ORs) and 95% confidence intervals (CIs); (3) full text in English or Chinese available. For duplicate reports, only the most complete one was included. Family-based association studies, case reports, case series, reviews, comments, letters, and conference presentations were excluded.

### Data extraction and quality assessment

The following data were extracted from included studies: (1) name of first author; (2) year of publication; (3) country and ethnicity of participants; (4) type of disease; (5) the number of cases and controls; and (6) genotypic distributions of *CTLA-4* gene polymorphisms in cases and controls. The probability value (*p* value) of Hardy–Weinberg equilibrium (HWE) test was also calculated.

The Newcastle–Ottawa scale (NOS) was used to assess the quality of eligible studies from three aspects: (1) selection of cases and controls; (2) comparability between cases and controls; and (3) exposure in cases and controls [16]. The NOS has a score range of 0–9, and studies with a score of more than 7 were assumed to be of high quality.

Two reviewers conducted data extraction and quality assessment independently. When necessary, the reviewers wrote to the corresponding authors for extra information. Any disagreement between two reviewers was solved by discussion until a consensus was reached.

### Statistical analyses

All statistical analyses in the present study were conducted with Review Manager Version 5.3.3 (The Cochrane Collaboration, Software Update, Oxford, United Kingdom). ORs and 95% CIs were used to assess potential associations of *CTLA-4* gene polymorphisms with the susceptibility to DM in dominant, recessive, over-dominant, and allele models, and a *P* value of 0.05 or less was considered to be statistically significant. Between-study heterogeneity was evaluated by *I*^2^ statistic. If *I*^2^ was greater than 50%, random-effect models (REMs) would be used for analyses due to the existence of significant heterogeneities. Otherwise, fixed-effect models (FEMs) would be employed for analyses. Subgroup analyses by ethnicity of participants were subsequently performed. Sensitivity analyses were carried out to test the stability of the results. Funnel plots were applied to evaluate possible publication biases.

## Results

### Characteristics of included studies

**Figure 1:**
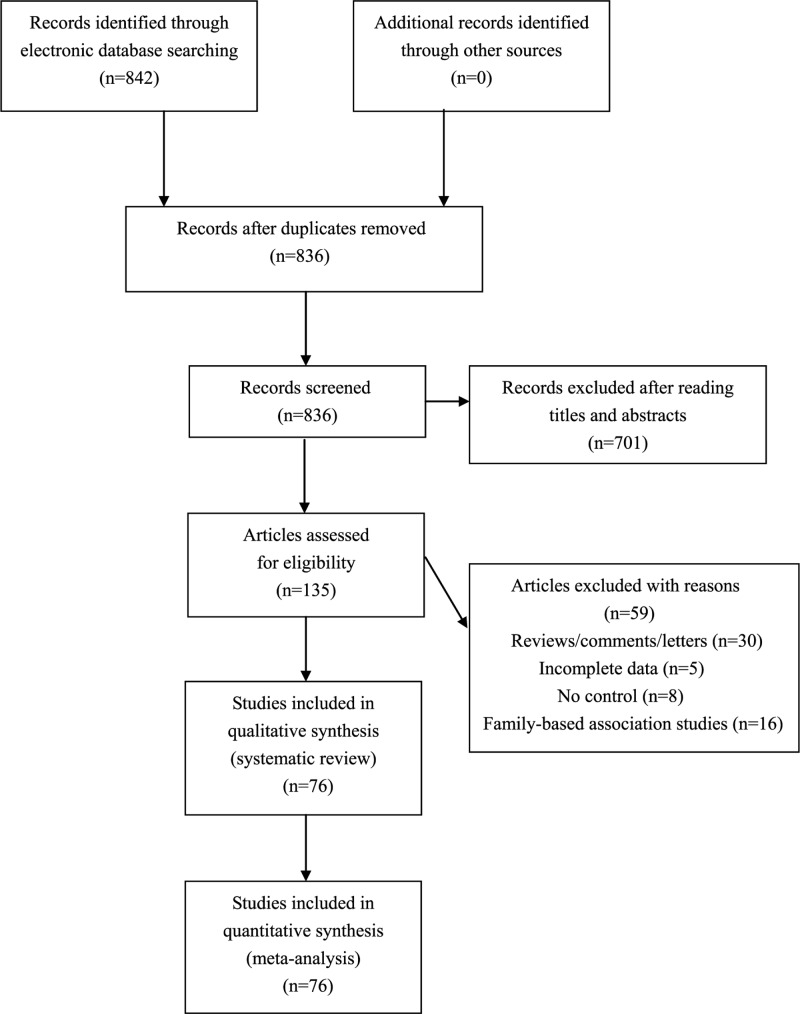
Flowchart of study selection for the present study

Our systematic literature search yielded 842 results. After exclusion of irrelevant and duplicate articles by reading titles and abstracts, 135 potentially relevant articles were retrieved for further evaluation. Another 59 articles were subsequently excluded after reading the full text. Finally, a total of 76 studies that met the inclusion criteria of our meta-analysis were included (see Figure 1). Characteristics of included studies are shown in Table 1.

**Table 1:** The characteristics of included studies

First author, year	Country	Ethnicity	Type of disease	Sample size	Genotypes (wtwt/wtmt/mtmt)		*P* value for HWE	NOS score
					Cases	Controls		
**rs231775 A/G**								
Abe 1999	Japan	East Asian	T1DM	111/445	50/45/16	177/207/61	0.969	7
Ahmadi 2013	Iran	South Asian	T1DM	60/107	25/32/3	67/36/4	0.757	7
Ahmedov 2006	Azerbaijan Republic	Caucasian	T1DM	160/271	80/58/22	143/103/25	0.307	7
Awata 1998	Japan	East Asian	T1DM	173/425	72/80/21	170/197/58	0.938	7
Balic 2009	Chile	Mixed	T1DM	300/310	125/136/39	138/131/41	0.267	7
Baniasadi 2006	India	South Asian	T1DM	130/180	50/62/18	76/79/25	0.541	8
Benmansour 2010	Tunisia	South Asian	T1DM	228/193	98/83/47	104/69/20	0.102	7
Bouqbis 2003	Morocco	Caucasian	T1DM	118/114	59/52/7	59/47/8	0.742	7
Caputo 2005	Argentina	Mixed	T1DM	186/168	76/84/26	71/76/21	0.924	7
Çelmeli 2013	Turkey	Caucasian	T1DM	91/99	38/40/13	43/49/7	0.161	7
Chen 2011	China	East Asian	T1DM	360/728	199/136/25	329/319/80	0.839	8
Cinek 2002	Czech Republic	Caucasian	T1DM	305/289	123/125/57	106/133/50	0.458	8
Cosentino 2002	Italy	Caucasian	T1DM	80/85	21/55/4	40/40/5	0.219	7
Dallos 2008	Slovakia	Caucasian	T1DM	171/231	33/72/66	55/126/50	0.164	8
Ding 2010	China	East Asian	T1DM	23/33	2/14/7	28/4/1	0.126	7
Djilali-Saiah 1998	France	Caucasian	T1DM	112/100	37/41/34	47/37/16	0.070	7
Donner 1997	Germany	Caucasian	T1DM	293/325	91/147/55	135/149/41	0.990	7
Douroudis 2009	Estonia	Caucasian	T1DM	170/230	45/79/46	68/125/37	0.104	7
Douroudis 2009	Finland	Caucasian	T1DM	404/725	69/203/132	159/378/188	0.232	7
Ei Wafai 2011	Saudi Arabia	South Asian	T1DM	39/46	9/21/9	25/21/0	0.045	7
Fajardy 2002	France	Caucasian	T1DM	134/273	41/76/17	96/146/31	0.027	7
Ferreira 2009	Brazil	Mixed	T1DM	49/48	26/20/3	22/21/5	0.997	7
Genc 2004	Turkey	Caucasian	T1DM	48/80	24/20/4	43/34/3	0.233	8
Haller 2007	Estonia	Caucasian	T1DM	131/252	27/62/42	77/135/40	0.131	7
Hauache 2005	Brazil	Mixed	T1DM	124/75	42/63/19	30/34/11	0.787	8
Hayashi 1999	Japan	East Asian	T1DM	117/141	54/42/21	72/47/22	0.005	7
Ide 2004	Japan	East Asian	T1DM	116/114	56/49/11	34/59/21	0.603	7
Ihara 2001	Japan	East Asian	T1DM	160/200	NA	NA	NA	7
Ikegami 2006	Japan	East Asian	T1DM	767/715	439/285/43	395/283/37	0.131	7
Jin 2015	China	East Asian	T1DM	402/482	182/194/26	169/241/72	0.354	7
Jung 2009	Korea	East Asian	T1DM	176/90	94/58/24	46/31/13	0.053	7
Kamoun 2001	Tunisia	South Asian	T1DM	74/49	32/38/4	11/28/10	0.316	7
Kawasaki 2008	Japan	East Asian	T1DM	91/369	48/36/7	122/186/61	0.484	7
Khoshroo 2017	Iran	South Asian	T1DM	39/40	11/10/18	13/15/12	0.114	7
Kikuoka 2001	Japan	East Asian	T1DM	125/200	57/62/6	78/88/34	0.287	8
Klitz 2002	USA	Mixed	T1DM	94/90	NA	NA	NA	7
Korolija 2009	Croatia	Caucasian	T1DM	102/193	48/36/18	96/84/13	0.345	7
Kumar 2015	India	South Asian	T1DM	232/305	95/101/36	169/116/20	0.987	7
Lee 2000	Taiwan	East Asian	T1DM	253/91	150/85/18	37/45/9	0.378	7
Lemos 2009	Portugal	Caucasian	T1DM	207/249	82/95/30	111/108/30	0.637	7
Liang 2004	Japan	East Asian	T1DM	29/40	19/10/0	10/27/3	0.013	7
Ma 2002	China	East Asian	T1DM	31/36	5/11/15	19/9/8	0.007	7
McCormack 2001	UK	Caucasian	T1DM	144/307	NA	NA	NA	7
Mochizuki 2003	Japan	East Asian	T1DM	97/60	44/36/17	21/27/12	0.539	7
Mojtahedi 2005	Iran	South Asian	T1DM	109/331	21/78/10	146/149/36	0.826	7
Momin 2009	USA	Mixed	T1DM	261/280	113/112/36	131/119/30	0.702	7
Mosaad 2012	Egypt	South Asian	T1DM	104/78	37/59/8	38/39/1	0.010	7
Nisticò 1996	Italy	Caucasian	T1DM	483/529	161/248/74	236/242/51	0.329	8
Ongagna 2002	France	Caucasian	T1DM	62/84	49/10/3	43/27/14	0.013	7
Osei-Hyiaman 2001	Japan	East Asian	T1DM	350/420	110/166/74	201/177/42	0.741	8
Padma-Malini 2018	India	South Asian	T1DM	196/196	78/93/25	128/61/7	0.936	8
Pérez 2009	Chile	Mixed	T1DM	260/255	116/110/34	110/106/39	0.115	7
Philip 2011	India	South Asian	T1DM	53/53	5/30/18	32/15/6	0.064	7
Ranjouri 2016	Iran	South Asian	T1DM	50/50	36/12/2	41/7/2	0.044	8
Saleh 2008	Egypt	South Asian	T1DM	396/396	166/175/55	215/150/31	0.501	7
Song 2012	China	East Asian	T1DM	108/100	73/25/10	45/39/16	0.138	7
Steck 2005	USA	Mixed	T1DM	102/198	NA	NA	NA	7
Takara 2000	Japan	East Asian	T1DM	74/107	16/25/33	34/43/30	0.044	7
Tavares 2015	Brazil	Mixed	T1DM	204/305	82/91/31	127/140/38	0.952	7
Van der Auwera 1997	Belgium	Caucasian	T1DM	525/530	NA	NA	NA	7
Wang 2002	China	East Asian	T1DM	90/84	13/54/23	32/42/10	0.500	7
Wang 2008	China	East Asian	T1DM	48/192	4/29/15	124/52/16	0.004	8
Wood 2002	Germany	Caucasian	T1DM	176/220	59/84/33	99/95/26	0.662	7
Xiang 2006	China	East Asian	T1DM	179/290	79/86/14	87/153/50	0.216	8
Yanagawa 1999	Japan	East Asian	T1DM	110/200	45/46/19	78/88/34	0.287	7
Yang 2006	China	East Asian	T1DM	34/71	23/8/3	32/28/11	0.253	7
Zalloua 2004	USA	Mixed	T1DM	190/102	91/75/24	53/45/4	0.137	7
Ahmadi 2013	Iran	South Asian	T2DM	56/107	35/18/3	67/36/4	0.757	7
Ding 2010	China	East Asian	T2DM	34/33	21/11/2	28/4/1	0.126	7
Gu 2007	China	East Asian	T2DM	111/39	35/71/5	15/20/4	0.475	7
Haller 2007	Estonia	Caucasian	T2DM	244/252	76/122/46	77/135/40	0.131	7
Jin 2015	China	East Asian	T2DM	330/482	128/171/31	169/241/72	0.354	7
Khoshroo 2017	Iran	South Asian	T2DM	71/40	39/17/18	13/15/12	0.114	7
Kiani 2016	Iran	South Asian	T2DM	111/100	60/42/9	41/39/20	0.066	7
Ma 2002	China	East Asian	T2DM	31/36	7/17/7	19/9/8	0.007	7
Rau 2001	Germany	Caucasian	T2DM	300/466	126/140/34	183/215/68	0.707	8
Shih 2018	Taiwan	East Asian	T2DM	278/287	118/127/33	101/150/36	0.084	7
Uzer 2010	Turkey	Caucasian	T2DM	72/169	43/24/5	113/45/11	0.035	7
Wang 2008	China	East Asian	T2DM	192/192	59/102/31	124/52/16	0.004	8
Yu 2006	China	East Asian	T2DM	121/39	35/71/5	15/20/4	0.475	7
**rs5742909**								
Almasi 2015	Iran	South Asian	T1DM	153/189	143/10/0	174/14/1	0.235	7
Balic 2009	Chile	Mixed	T1DM	300/310	243/50/7	253/47/10	<0.001	7
Baniasadi 2006	India	South Asian	T1DM	130/180	113/15/2	170/10/0	0.701	8
Benmansour 2010	Tunisia	South Asian	T1DM	228/193	159/52/17	156/29/8	<0.001	7
Bouqbis 2003	Morocco	Caucasian	T1DM	118/114	106/12/0	110/4/0	0.849	7
Caputo 2007	Argentina	Mixed	T1DM	178/136	149/28/1	110/26/0	0.218	7
Chen 2011	China	East Asian	T1DM	359/728	281/71/7	550/164/14	0.664	8
Douroudis 2009	Estonia	Caucasian	T1DM	61/230	52/8/1	178/49/3	0.857	7
Ihara 2001	Japan	East Asian	T1DM	160/200	NA	NA	NA	7
Lee 2001	Taiwan	East Asian	T1DM	347/260	303/42/2	201/56/3	0.681	7
Saleh 2008	Egypt	South Asian	T1DM	396/396	180/178/38	214/164/18	0.053	7
Steck 2005	USA	Mixed	T1DM	102/198	NA	NA	NA	7
Wang 2008	China	East Asian	T1DM	48/189	30/18/0	155/34/0	0.174	8
Zouidi 2014	Tunisia	South Asian	T1DM	76/162	68/7/1	145/15/2	0.040	7
Kiani 2016	Iran	South Asian	T2DM	111/100	75/26/10	88/10/2	0.020	7
Shih 2018	Taiwan	East Asian	T2DM	278/287	227/49/2	215/67/5	0.933	7
Uzer 2010	Turkey	Caucasian	T2DM	72/169	55/14/3	116/43/10	0.036	7
Wang 2008	China	East Asian	T2DM	192/189	157/35/0	155/34/0	0.174	8

*Abbreviations*: wt, wild type; mt, mutant type; NA, not available.

### CTLA-4 gene polymorphisms and the susceptibility to DM

Significant associations with susceptibility to T1DM were detected for rs231775 (dominant model: *P*=0.008, OR = 0.83, 95%CI 0.73–0.95; recessive model: *P*=0.003, OR = 1.27, 95%CI 1.09–1.50; allele model: *P*=0.004, OR = 0.85, 95%CI 0.77–0.95) and rs5742909 (recessive model: *P*=0.02, OR = 1.50, 95%CI 1.05–2.13) polymorphisms in overall population. Nevertheless, no any positive results were detected for T2DM in overall population.

Further subgroup analyses revealed that rs231775 polymorphism was significantly associated with susceptibility to T1DM in Caucasians (dominant, recessive, and allele models) and South Asians (dominant, recessive, over-dominant, and allele models), but not in East Asians. Moreover, rs231775 polymorphism was also significantly associated with susceptibility to T2DM in East Asians (over-dominant model) and South Asians (recessive and allele models), but not in Caucasians. Additionally, we also found that rs5742909 polymorphism was significantly associated with susceptibility to T1DM in South Asians (dominant, recessive, over-dominant, and allele models), but not in East Asians and Caucasians (see Table 2).

**Table 2:** Overall and subgroup analyses for *CTLA-4* gene polymorphisms and DM

Variables	Sample size	Dominant comparison		Recessive comparison		Over-dominant comparison		Allele comparison	
		*P* value	OR (95%CI)	*P* value	OR (95%CI)	*P* value	OR (95%CI)	*P* value	OR (95%CI)
**rs231775 A/G**									
*T1DM*									
Overall	11420/14674	0.008^*^	0.83 (0.73–0.95)	0.003^^*^^	1.27 (1.09–1.50)	0.59	1.03 (0.93–1.13)	0.004^*^	0.85 (0.77–0.95)
Caucasian	3854/5102	<0.0001^†^	0.74 (0.67–0.81)	<0.0001^†^	1.61 (1.42–1.83)	0.76	0.99 (0.90–1.08)	<0.0001^†^	0.77 (0.72–0.82)
East Asian	4024/5633	0.73	1.05 (0.80–1.37)	0.78	0.95 (0.69–1.32)	0.32	0.92 (0.77–1.09)	0.79	1.03 (0.83–1.28)
South Asian	1710/2024	<0.0001^†^	0.52 (0.38–0.70)	0.005^*^	1.79 (1.19–2.70)	0.001^*^	1.47 (1.17–1.86)	<0.0001^†^	0.60 (0.48–0.75)
*T2DM*									
Overall	1951/2242	0.34	0.85 (0.61–1.19)	0.12	1.16 (0.96–1.40)	0.14	1.22 (0.94–1.59)	0.58	0.94 (0.74–1.19)
Caucasian	616/887	0.82	1.03 (0.83–1.27)	0.75	0.95 (0.70–1.29)	0.99	1.00 (0.81–1.23)	0.75	1.03 (0.88–1.20)
East Asian	1097/1108	0.08	0.58 (0.32–1.07)	0.59	0.88 (0.54–1.42)	0.04^‡^	1.66 (1.03–2.68)	0.15	0.74 (0.49–1.12)
South Asian	238/247	0.06	0.59 (0.34–1.02)	0.02^‡^	1.56 (1.08–2.27)	0.36	0.84 (0.57–1.23)	0.003^*^	0.65 (0.49–0.87)
**rs5742909 C/T**									
*T1DM*									
Overall	2656/3485	0.37	0.87 (0.65–1.18)	0.02^‡^	1.50 (1.05–2.13)	0.51	1.10 (0.83–1.45)	0.36	0.89 (0.70–1.13)
Caucasian	179/344	0.77	0.78 (0.15–3.96)	0.84	1.26 (0.13–12.34)	0.80	1.25 (0.23–6.72)	0.72	0.76 (0.17–3.36)
East Asian	914/1377	0.99	1.00 (0.47–2.14)	0.74	0.87 (0.38–1.98)	1.00	1.00 (0.47–2.13)	0.80	1.07 (0.65–1.73)
South Asian	983/1120	0.0004^§^	0.68 (0.55–0.84)	0.002^§^	2.05 (1.30–3.23)	0.04^‡^	1.27 (1.02–1.58)	<0.0001^†^	0.69 (0.58–0.82)
*T2DM*									
Overall	653/745	0.80	0.92 (0.48–1.77)	0.89	1.11 (0.27–4.65)	0.93	1.02 (0.61–1.73)	0.76	0.90 (0.47–1.74)
East Asian	470/476	0.13	1.28 (0.93–1.75)	0.29	0.41 (0.08–2.12)	0.20	0.81 (0.59–1.12)	0.11	1.27 (0.95–1.71)

* *P* < 0.01.

† *P* < 0.0001.

‡ *P* < 0.05.

§ *P* < 0.001.

### Sensitivity analyses

Sensitivity analyses were carried out to test the stability of meta-analysis results by eliminating studies that deviated from HWE. No changes of results were detected for investigated *CTLA-4* gene polymorphisms in any comparisons, which indicated that our findings were quite statistically reliable.

### Publication biases

Potential publication biases in the present study were evaluated with funnel plots. No obvious asymmetry of funnel plots was observed in any comparisons, which suggested that our findings were unlikely to be impacted by severe publication biases.

## Discussion

Despite enormous advancements in pharmacotherapy over the past few decades, DM and its associated vascular complications are still leading causes of death and disability all over the world [17,18]. To date, the exact cause of DM is still largely unclear in spite of extensive investigations. However, the obvious familial aggregation tendency of DM indicated that genetic factors may significantly contribute to its occurrence and development [19]. Thus, identify potential genetic biomarkers is of particularly importance for an early diagnosis and a better prognosis of DM patients.

Previous studies showed that interferon α and its associated pathways could induce autoantigen presentation, active autoreactive monocytes, cytotoxic T-lymphocytes and NK cells, elicit endoplasmic reticulum stress of human islet B cells, and impair insulin production [20,21]. These results indicated that autoimmunity might result in destruction of islet B cells, contribute to less insulin production, and give rise to the development of DM. As far as we know, this is so far the most comprehensive meta-analysis about *CTLA-4* gene polymorphisms and DM, and our pooled analyses revealed that rs231775 and rs5742909 polymorphisms may serve as genetic biomarkers of T1DM, and rs231775 polymorphism may also serve as a genetic biomarker of T2DM. The stabilities of synthetic results were evaluated by sensitivity analyses, and no alterations of results were observed in any comparisons, which suggested that our findings were statistically stable. As for evaluation of heterogeneities, significant heterogeneities were detected for rs231775 polymorphism in every comparison of overall analyses for T1DM, and thus all analyses were performed with REMs. But in further subgroup analyses, a reduction tendency of heterogeneity was found in South Asians, which suggested that differences in ethnicity could partially explain observed heterogeneities between studies.

There are several points that need to be addressed about the present study. First, our findings indicated that rs231775 and rs5742909 polymorphisms could be used to identify individuals at higher risk of developing T1DM, and rs231775 polymorphism could also be used to identify individuals at higher risk of developing T2DM. There are two possible explanations for our positive findings. First, rs231775 and rs5742909 polymorphisms of the *CTLA-4* gene may lead to alternations in gene expression or changes in CTLA-4 protein structure, which may subsequently affect biological functions of CTLA-4, result in immune dysfunction and ultimately impact individual susceptibility to DM, especially T1DM. Second, it is noteworthy that several analyses were still based on limited number of studies, and therefore, further replication studies, especially in T2DM are still warranted to confirm these findings. Third, the pathogenic mechanism of DM is extremely complex, and hence despite our positive findings, it is unlikely that a single genetic polymorphism could significantly contribute to its development [22,23]. Fourth, due to lack of raw data, we failed to explore possible interactions of investigated *CTLA-4* gene polymorphisms. But to better illustrate the potential associations of *CTLA-4* gene polymorphisms with DM, we strongly recommend further studies to perform haplotype analyses and explore potential gene–gene interactions.

Our meta-analysis certainly has some limitations. First, although the general methodology qualities of included studies were good, it should be noted that we did not have access to genotypic distributions of investigated polymorphisms according to base characteristics of study subjects. Therefore, our results were derived from unadjusted estimations, and failure to conduct further adjusted analyses for baseline characteristics of participants such as age, gender, and co-morbidity conditions may influence the authenticity of our findings [24]. Second, significant heterogeneities were detected in certain subgroup comparisons, which indicated that the inconsistent results of included studies could not be fully explained by differences in ethnic background, and other unmeasured characteristics of participants may also partially attribute to between-study heterogeneities [25]. Third, associations between *CTLA-4* gene polymorphisms and DM may also be influenced by gene–environmental interactions. However, the majority of studies did not consider these potential interactions, which impeded us to perform relevant analyses accordingly [26]. Fourth, since only published articles were eligible for analyses, although funnel plots revealed no obvious publication biases, we still could not rule out the possibility of potential publication biases. Taken these limitations into consideration, the results of the present study should be interpreted with caution.

In conclusion, our findings indicated that rs231775 and rs5742909 polymorphisms may serve as genetic biomarkers of T1DM, and rs231775 polymorphism may also serve as a genetic biomarker of T2DM. Further well-designed studies, especially in T2DM are still warranted to confirm our findings, and future investigations also need to explore possible roles of other *CTLA-4* gene polymorphisms in DM.

## Abbreviations

CI: confidence interval
CTLA-4: cytotoxic T-lymphocyte-associated antigen 4
DM: diabetes mellitus
HWE: Hardy–Weinberg equilibrium
NOS: Newcastle–Ottawa scale
OR: odds ratio
REM: random-effect model
T1DM: type 1 diabetes mellitus
T2DM: type 2 diabetes mellitus
